# Subjective sleep quality, but not objective sleep measures, mediates the relationship between pre‐sleep worrying and affective wellbeing

**DOI:** 10.1111/jsr.14467

**Published:** 2025-01-30

**Authors:** Anika Werner, Justin Hachenberger, Kai Spiegelhalder, Jana‐Elisa Rueth, Angelika A. Schlarb, Arnold Lohaus, Sakari Lemola

**Affiliations:** ^1^ Faculty of Psychology and Sports Science, Department of Psychology Bielefeld University Bielefeld Germany; ^2^ Department of Psychiatry and Psychotherapy Medical Center ‐ University of Freiburg, Faculty of Medicine University of Freiburg Freiburg Germany

**Keywords:** affective wellbeing, ambulatory assessment, intensive longitudinal data, pre‐sleep worrying, sleep

## Abstract

Pre‐sleep worrying is associated with sleep disturbance, which in turn is associated with impaired affective wellbeing. However, studies examining the fine‐grained temporal order of these variables are still lacking. In particular, within‐person mediation of the association between pre‐sleep worrying and the following day's affective wellbeing by subjective and objective indicators of sleep has not been tested yet. This study investigates the extent to which pre‐sleep worrying predicts positive/negative affect the following day, and whether subjective/objective sleep disturbances are possible mediators for this relationship. Data from two experience sampling studies were pooled for the analyses, resulting in a total sample of 220 participants aged between 18 and 30 years (*M* = 23.2 years, SD = 2.8). The hypotheses were tested at both the between‐ and within‐subject level using causal mediation analysis. The within‐subject analyses revealed partial mediation of the relationship between pre‐sleep worrying and positive as well as negative affect the next day by subjective sleep quality. By contrast, sleep as measured by actigraphy appears not to be relevant for the link between pre‐sleep worrying and affective wellbeing the following day. Baseline levels of depressive symptoms and sleep disturbances did not moderate the associations between pre‐sleep worrying, sleep indices and affective states the following day. Improving perceived sleep quality by addressing pre‐sleep worrying could be a potential avenue to enhance affective wellbeing and promote better mental health in young adults.

## 1 INTRODUCTION

Poor affective wellbeing is an important public health concern (Trudel‐Fitzgerald et al., 2019), and establishing a better understanding of the mechanisms leading to poor affective wellbeing is essential to design effective prevention and treatment approaches. On the one hand, there is a large body of evidence that sleep disturbance is associated with poor affective wellbeing and actually predates the onset of mental health disorders (Freeman et al., 2020). On the other hand, dysfunctional cognition, and in particular worrying and rumination, are thought to play a key role for both the development of poor affective wellbeing and sleep disturbance (Clancy et al., 2020; Merolla et al., 2019). The aim of the present work is to examine the links between pre‐sleep worrying, sleep disturbance and affective wellbeing, and in particular to test a mediation model stipulating sleep disturbance as a mediator between pre‐sleep worrying and poor affective wellbeing based on data from two intensive longitudinal studies allowing for the inspection of fine‐grained temporal ordering. The following section will introduce the underpinning evidence of and mechanisms involved in the hypothesized mediation model. In particular, this includes evidence about: (a) the mechanisms linking impaired sleep quality with poor affective wellbeing; and (b) the mechanisms linking pre‐sleep worrying with both impaired sleep quality and poor affective wellbeing.

### 1.1 Mechanisms linking sleep quality and affective wellbeing

Poor subjective sleep quality is associated with poor affective wellbeing the following day, including decreased positive affect and increased negative affect (Das‐Friebel et al., 2020; Hachenberger et al., 2023; Konjarski et al., 2018; Lenneis et al., 2024). Depressive and anxiety disorders that are closely linked to affect (Werner‐Seidler et al., 2013) are highly comorbid with insomnia, and meta‐analyses indicated that the onset of depression and anxiety disorders is often secondary to the onset of insomnia (Hertenstein et al., 2019). However, the mechanism by which sleep is linked to affective wellbeing is less well understood.

One possible mechanistic model suggests lack of sleep involving short total sleep time (TST) as a causal factor for negative affect and poorer emotion regulation (Baum et al., 2014; Yoo et al., 2007). A large number of experimental studies (see Palmer et al., 2023, for a meta‐analysis) have confirmed that lack of sleep causes a decrease in positive affect and an increase in negative affect, general mood disturbances, negative emotional reactivity, anxiety symptoms and depressive symptoms (Palmer et al., 2023). Neuroimaging studies suggest that sleep restriction increases amygdala activation and leads to a functional disconnect between the prefrontal cortex and the amygdala (Yoo et al., 2007), which was interpreted as the cause of increased negative affect due to increased excitability by negative or ambivalent stimuli and a decreased prefrontal control of negative affect, respectively.

While intuitively appealing, some observations are difficult to reconcile with the proposition that lack of sleep is the driving factor behind the negative effects of impaired sleep on affective wellbeing. First, insomnia symptoms are not very strongly correlated with objectively measured sleep parameters involving, for example, low levels of sleep efficiency (SE) and short TST (Harvey & Tang, 2012; Lemola, Ledermann, & Friedman, 2013; Lemola, Räikkönen, et al., 2013). Second, cognitive behavioural treatment of insomnia (CBT‐I), which very efficiently decreases insomnia symptoms and eventually also leads to improved mental health in general (Freeman et al., 2017), actually does not increase objectively measured TST in adults (Mitchell et al., 2019). Taking these observations together, it is unclear whether and how strongly shortened sleep is actually a mechanism involved in the link between sleep disturbance and poor affective wellbeing. It is therefore possible that aspects of subjective sleep quality that are not reflected in objective sleep measures play a more important role when considering affective wellbeing in connection with sleep.

### 1.2 Mechanisms linking dysfunctional cognition with sleep and affective wellbeing

Dysfunctional cognitions are another construct that is closely linked to sleep and affective wellbeing. The hyperarousal model of insomnia stipulates that both increased cognitive arousal (including worrying and rumination) as well as neurophysiological and neuroendocrine arousal are playing a key role for the aetiology and course of insomnia (Riemann et al., 2010). Recent work has particularly shown the outstanding role of cognitive arousal for insomnia, including the inability to suppress mental activities such as worrying and rumination while trying to fall asleep (Kalmbach et al., 2020; Pillai & Drake, 2015; Yeh et al., 2015). Increased cognitive arousal is associated with poor subjective and objective sleep parameters, for example decreased sleep continuity as represented by lower SE and shorter sleep duration as represented by short TST in healthy sleepers and people with insomnia (Kalmbach et al., 2020), with worrying and rumination being particularly important for nighttime sleep disturbances and daytime impairment including decreased affective wellbeing. Cognitive arousal therefore appears to precede sleep problems. In particular, worrying about one's own sleep and daytime consequences may further increase and maintain sleep problems, leading into a vicious circle (Yang et al., 2011). While the causal link between pre‐sleep cognitive arousal and sleep disturbances is likely bi‐directional (Kalmbach et al., 2020), it has also been suggested that sleep disturbance is a mediator of the association between cognitive arousal and symptoms of depression (Clancy et al., 2020). In line with that, insomnia partly mediates the effect of pre‐sleep cognitive arousal on depression in perinatal women (Kalmbach et al., 2021). Therefore, a mediating effect of sleep disturbances for the association between cognitive arousal and affective wellbeing is also conceivable.

### 1.3 Gap in research

Although there are already numerous studies on the relationship between pre‐sleep worrying, sleep and affective wellbeing, there is a lack of intensive longitudinal studies allowing separate analysis of within‐subject and between‐subject variability. However, such research is needed to shed more light on the fine‐grained temporal sequence of within‐subject processes, which is of particular importance as there is growing evidence that the processes producing within‐subject variability often differ fundamentally from the processes leading to between‐subject variability (Fisher et al., 2018; Hamaker, 2012; Hamaker & Wichers, 2017).

To date, there are only a few intensive longitudinal studies that consider pre‐sleep worrying, sleep and/or affective wellbeing. Two studies focusing on within‐subject processes showed that higher state levels of pre‐sleep rumination predict an increase in subsequent objective and subjective sleep‐onset latency (Pillai et al., 2014; Sladek et al., 2020). Further studies showed that better subjective sleep quality predicts higher levels of positive affect the subsequent day (Das‐Friebel et al., 2020; Hachenberger et al., 2023; Kalmbach et al., 2014; Lenneis et al., 2024). However, only one study examined the three variables (pre‐sleep worrying, sleep, and affective wellbeing) together, showing that negative repetitive thoughts in the evening predict a longer sleep‐onset latency, and decreased SE predicts lower levels of positive affect the following morning (Takano et al., 2014). Thus, a mediating role of sleep parameters for the relationship between dysfunctional cognitions and positive/negative affect the next day is conceivable.

### 1.4 Aims of the current study

First, the present study examines whether subjective sleep quality as reported the following morning mediates the association between pre‐sleep worrying and positive affect (Research Question 1a; RQ1a) as well as negative affect on the following day (Research Question 1b; RQ1b). Furthermore, objective nocturnal sleep parameters (i.e. actigraphy‐derived SE and TST as indicators of sleep continuity and sleep duration, respectively) will be examined separately as potential mediators of the link between pre‐sleep worrying and positive affect (Research Question 2a; RQ2a) as well as negative affect the next day (Research Question 2b; RQ2b). TST and SE were selected as variables here because previous studies have shown that increased cognitive arousal was associated with lower levels of TST and SE (Kalmbach et al., 2020), and that lower levels of TST and SE were in turn relatively consistently associated with poor affect (Hickman et al., 2024), suggesting a possible mediating pathway. Furthermore, TST and SE are commonly analysed actigraphic sleep measures, which have also been consistently defined and operationalized in previous studies (Astill et al., 2012; Konjarski et al., 2018). All mediation models are tested both on the within‐ and between‐subject level. Second, we also examine moderation by baseline levels of depressive symptoms and sleep disturbance representing insomnia symptoms (Research Question 3; RQ3) as the associations may differ between individuals with higher or lower levels of depressive and insomnia symptoms (Mor & Winquist, 2002; Takano & Tanno, 2011).

Sleep disturbances are common, and insomnia as a symptom occurs in up to one‐third of the general population (Ohayon, 2002). Therefore, the research questions presented will be analysed in a sample of the general population, allowing for a broader understanding of how pre‐sleep worrying relates to sleep and affective wellbeing at different levels of sleep disturbance severity by testing its moderating role. Furthermore, the observation of these mechanisms of action in the general population may also allow the prevention of sleep problems.

## 2 METHODS

### 2.1 Design and procedure

Data from two studies using an experience sampling methodology (ESM) were used to answer the research questions of the present study. Both studies were approved by the Ethics Committee of Bielefeld University (reference numbers 2022‐063 and 2022‐280). Data were collected between April and July 2022 for Study 1, and between January 2023 and February 2024 for Study 2. Participants were recruited online through social media, email distribution lists from various German universities, and the study participation management portal of the Department of Psychology at Bielefeld University. When registering for the study, participants were informed about the procedure and conditions of study participation as well as their rights (e.g. regarding data protection and withdrawal from the study). All participants provided informed consent and confirmed that they met the following inclusion criteria: (a) age 18–29 years; (b) being fluent in German; and (c) having a smartphone with an Android operating system available for the duration of the study (alternatively participants could receive a lab phone for participation).

Data collection lasted 29 days for each batch in both studies. On the first day, participants completed a baseline assessment. ESM surveys started on the second day using the movisensXS app (movisens GmbH, Karlsruhe, Germany). Participants were asked to complete short surveys seven times per day in Study 1, and three times a day in Study 2 for 28 consecutive days. Additionally, participants wore accelerometers (GENEActiv, Activinsights Ltd, Kimbolton, UK) on their non‐dominant wrist during the ESM period. Participants were informed that they could take off the accelerometer if necessary (e.g. when showering). However, participants were encouraged to wear it as much as possible.

**FIGURE 1:**
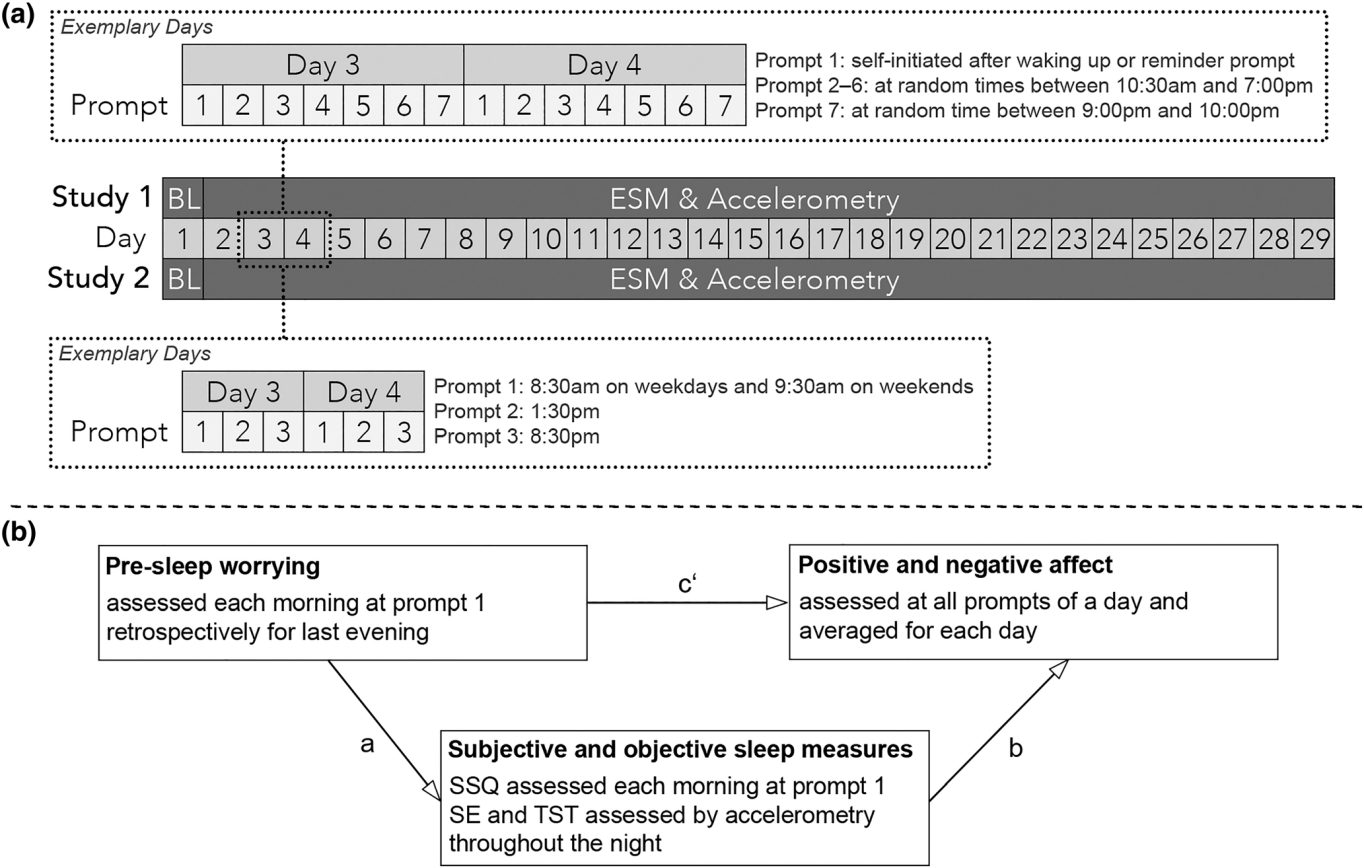
Study design and conceptual illustration of the mediation analyses. BL, baseline assessment; ESM, experience sampling methodology; SSQ, subjective sleep quality; SE, sleep efficiency; TST, total sleep time. (a) The relevant details of the design in both studies with exemplary presentation of Days 3 and 4 (can be generalized to all other days). (b) The conceptual idea behind the mediation analyses.

In Study 1, participants were able to manually start the first survey of each day, and were asked to do so as soon as possible after waking up. If participants did not start the first survey manually, they received a reminder prompt between 08:00 hours and 09:00 hours on weekdays, and between 09:00 hours and 10:00 hours on weekends. For the next five surveys during the day, prompts were sent out between 10:30 hours and 19:00 hours. Each day's last prompt was sent out between 21:00 hours and 22:00 hours on weekdays and weekends. All prompts were sent out at random times within the respective time interval. Also, there was a gap of at least 90 min between two adjacent prompts. Participants could respond to the surveys within 30 min after receiving the prompts. In Study 2, participants received prompts for the first survey of a day at 08:30 hours on weekdays and 09:30 hours on weekends. The prompts for the second and third surveys were sent out at 13:30 hours and 20:30 hours, respectively. Participants could respond to the surveys within 60 min after receiving the prompts. In both studies, a survey would be automatically marked as missing when participants did not respond. Participants were instructed to ignore prompts in situations that could cause danger to themselves or others (e.g. while driving). The details of both studies are shown in Figure 1.

### 2.2 Participants

Overall, 230 participants took part in both studies, of which 10 participants did not have a single night of complete data (i.e. the accelerometry and/or morning questionnaire was missing) and were therefore excluded from this analysis. Accordingly, the final sample consisted of 220 individuals (82.7% female; 14.5% male; 2.7% preferred not to report their gender). The mean age was 23.2 years (SD = 2.8; range 18–30 years). Most participants were university students (92.7%). Of the 5940 possible nights, participants provided complete data (complete first survey of each day and valid accelerometry data for the night) for a total of 4255 nights (71.6%). On average, complete data of 19.3 nights (SD = 6.1) was available per participant. Separate sample descriptions for both studies are presented in Table 1.

**TABLE 1:** Demographics and descriptive statistics.

	Study 1 (*N* = 129)	Study 2 (*N* = 91)	Pooled data (*N* = 220)	
	*M* (SD)/*n* (%)	*M* (SD)/*n* (%)	*M* (SD)/*n* (%)	Observed min–max
Demographics				
Gender				
Female	107 (82.9)	75 (82.4)	182 (82.7)	–
Male	20 (15.5)	12 (13.2)	32 (14.5)	–
Preferred not to answer	2 (1.6)	4 (4.4)	6 (2.7)	–
Age (years)	23.7 (2.8)	22.4 (2.8)	23.2 (2.8)	18–30
Baseline				
Depressive symptoms (PHQ‐9)	8.0 (4.7)	8.3 (5.4)	8.2 (5.0)	0–27
No–mild symptoms	87 (67.4)	61 (67.0)	148 (67.3)	–
Moderate–severe symptoms	42 (32.6)	30 (33.0)	72 (32.7)	–
Insomnia symptoms^a^	6.2 (3.1)^b^	9.2 (5.8)	–^c^	–^c^
Good sleepers	56 (43.8)^b^	41 (45.1)	125 (57.1)^b^	–
Bad sleepers	72 (56.2)^b^	50 (54.9)	94 (42.9)^b^	–
ESM				
Nights with complete data	19.2 (6.3)	19.6 (5.7)	19.3 (6.1)	2–27
Pre‐sleep worrying	28.0 (27.6)	32.1 (28.7)	29.7 (28.1)	0.0–100.0
Subjective sleep quality	59.0 (25.0)	56.2 (25.6)	57.8 (25.3)	0.0–100.0
Positive affect	165.2 (48.0)	152.3 (49.5)^d^	159.8 (49.0)	0.0–298.8
Negative affect	67.7 (48.0)	75.7 (52.8)	71.0 (50.2)	0.0–271.0
Accelerometry				
SE (%)	78.1 (11.1)	77.3 (11.7)	77.8 (11.4)	1.5–98.4
TST (hr)	6 (1.4)	6.1 (1.5)	6 (1.4)	0.1–12.9

Abbreviations: ESM, experience sampling methodology; PHQ‐9, Patient Health Questionnaire‐9; SE, sleep efficiency; TST, total sleep time.

^a^ Measured with the PSQI in Study 1, and with the ISI in Study 2.

^b^ PSQI data of one participant were missing (Study 1).

^c^ Data not shown, because different questionnaires were used in both studies and scores were *z*‐standardized within each study before pooling and analysis.

^d^ Value significantly differs from Study 1, *p* < 0.05.

[Correction added on 19 February 2025, after first online publication: In the last column of “Accelerometry” in Table 1, the values for “SE%” and “TST (hr)” have been interchanged."]

### 2.3 Measures and instruments

The instruments used to measure the relevant constructs and variables were mostly identical in both studies, and are therefore described together. Any differences between the two studies are explicitly mentioned.

#### 2.3.1 Time‐invariant (baseline) variables

##### Depressive symptoms

Depressive symptoms were measured at baseline with the Patient Health Questionnaire‐9 (PHQ‐9; Kroenke et al., 2001; Löwe et al., 2002). The participants indicated on a four‐point scale (0 = *not at all*; 1 = *several days*; 2 = *more than half the days*; 3 = *nearly every day*) how often nine depressive symptoms occurred in the preceding 2 weeks. Out of all nine items, a sum score was computed (0–27 points). Individuals with scores below 10 were considered as having no to mild symptoms. Individuals with scores of 10 and higher were considered as having moderate to severe symptoms (Kroenke et al., 2001). The internal consistency of the subscale was *α* = 0.84 in the pooled data of both studies.

##### Sleep disturbances

Sleep disturbances representing insomnia symptoms were measured at baseline with the Pittsburgh Sleep Quality Index (PSQI; Buysse et al., 1989) in Study 1 and with the Insomnia Severity Index (ISI; Bastien, 2001; Gerber et al., 2016) in Study 2. The PSQI is a 19‐item, self‐rated questionnaire designed to measure subjective sleep quality in the past month. The 19 items are divided into seven components, including sleep duration, sleep disturbance, sleep latency, disruption of daytime functioning due to sleepiness, SE, overall sleep quality and use of sleep medications. Each of the sleep components results in a score between 0 and 3. The values of the sleep components are summed together to a total score (0–21 points), with a higher score indicating poorer sleep quality/more sleep disturbances (Buysse et al., 1989). The ISI is composed of seven items that evaluate the severity of sleep disturbance during the past 2 weeks. Each item is rated on a five‐point Likert scale, and the sum total score indicates the severity of insomnia. The internal consistency of the ISI was *α* = 0.86 in the current study. Individuals with scores below 6 on the PSQI or below 8 on the ISI (no insomnia symptoms) were considered as good sleepers. Individuals with scores of 6 or higher on the PSQI, or 8 or higher on the ISI (at least subthreshold insomnia) were considered as bad sleepers (Bastien, 2001; Buysse et al., 1989). Before the data of both studies were pooled, the PSQI and ISI were *z*‐standardized within the respective study.

#### 2.3.2 Time‐variant (ESM) variables

##### Positive and negative affect

The measurement of affect was based on Das‐Friebel et al. (2020). Positive affect was measured with the items content, enthusiastic and happy. Negative affect was measured with the items sad, upset and worried. In each ESM survey, the participants were asked to indicate on a visual analogue scale to what extent they felt each affective state (“How … do you feel at the moment?”, 0 = *not at all*, 100 = *very much*). Sum scores for positive and negative affect were computed for each prompt and then averaged across each day. Higher scores indicated higher levels of positive and negative affect, respectively. The internal consistencies across all measurement points were *ω*_between_ = 0.94 and *ω*_within_ = 0.88 for positive affect, and *ω*_between_ = 0.90 and *ω*_within_ = 0.74 for negative affect in the pooled data.

##### Pre‐sleep worrying

Pre‐sleep worrying was measured in the first questionnaire of each day. Participants were asked to indicate on a visual analogue scale (0 = *not at all*, 100 = *very much*) to what extent they were worried or upset before sleep (“Were you worried or upset about something before you went to sleep last night?”).

##### Subjective sleep quality

Subjective sleep quality was also assessed in the first questionnaire of each day. The participants were asked to indicate on a visual analogue scale how satisfied they were with their sleep the previous night (“How satisfied are you with your sleep last night?”, 0 = *not at all*, 100 = *very much*), which was adapted for the use in intensive longitudinal studies from an item validated to measure subjective sleep quality (Ohayon & Paiva, 2005; Ohayon & Partinen, 2002; Ohayon & Zulley, 2001; Yu et al., 2012).

#### 2.3.3 Accelerometer‐assessed sleep indicators

The TST and SE were derived from the acceleration data measured by the triaxial accelerometers (GENEActiv, Activinsights Ltd, Kimbolton, UK). A sampling rate of 60 Hz was used in both studies. The raw data were processed with the R‐package GGIR (Version 3.0‐9; Migueles et al., 2019) applying auto‐calibration (van Hees et al., 2014) and sleep detection (van Hees et al., 2015). The sleep detection algorithm was supplied with sleep diary data (sleep and wake‐up times), which were used as boundaries. In the first survey of each day, participants reported about what time they went to sleep (“When did you turn off the light and go to sleep?”) and at what time they woke up in the morning (“What time did you wake up this morning?”).

### 2.4 Statistical analyses

Data pre‐processing and all statistical analyses were performed using R (Version 4.4.0; R Core Team, 2024). The analysis scheme described below was carried out for the pooled data from both datasets and, in addition, for both datasets separately. Only the results of the pooled data are reported in the Results section. The results of the separate analyses for the datasets are presented in Tables S1–S9.

For the within‐subject analyses, all time‐variant variables (level 1) were within‐subject standardized, and all time‐invariant variables (level 2) were grand‐mean standardized. For the between‐subject analyses, we first averaged the time‐variant variables for each subject. The averages of the time‐variant variables and the time‐invariant variables were then grand‐mean standardized. To address the issue of alpha error accumulation in multiple comparisons, all *p*‐values (overall 276 values per dataset) were adjusted using false discovery rate (Benjamini & Hochberg, 1995). Only nights with complete data (i.e. valid accelerometry data for the night and completed morning questionnaire) were considered in the analyses.

To test RQ1 and RQ2, a mediation analysis was performed using R‐package *mediation* (Version 4.5.0; Tingley et al., 2014). For a model‐based causal mediation analysis, two models need to be specified: (1) a mediator model that includes the mediator variable (subjective sleep quality or objective sleep parameters) as the outcome and the independent variable (pre‐sleep worrying) as a predictor to test path a (Figure 1); and (2) an outcome model that includes the dependent variable (positive or negative affect) as the outcome and the independent variable (pre‐sleep worrying) and mediator variable (subjective sleep quality or objective sleep parameters) as predictors to test paths b and c' (Figure 1). These models were fitted using the lmer‐function of R‐package *lme4* (Version 1.1–35.1; Bates et al., 2015) for within‐subject analyses (multilevel models) and the lm‐function of base R for between‐subject analyses (linear regression). Gender and age as well as the respective other sleep measures (e.g. subjective sleep quality and TST when SE was tested as the mediator) were included as covariates in all models. An effect‐coded variable indicating which of the two studies a data point originated from was included as an additional covariate in the pooled data analyses. In the multilevel models for the within‐subject analyses, we included a fixed intercept and random slope for the main predictor and used restricted maximum likelihood estimation. Then we used the mediation‐function (Tingley et al., 2014) with the fitted models to estimate the average direct effects (ADE; i.e. path c'), average causal mediation effects (ACME; i.e. the indirect effect or paths a and b) and total effects.

To test the moderations of RQ3, the same analysis procedure was used as above, with the addition that the models for the mediator and the dependent variable also included a moderation term with the corresponding time‐invariant moderator variable (baseline depressive symptoms or sleep disturbances). Mediation analyses to contrast different value ranges of the moderator (–1 SD versus +1 SD) were only calculated if the moderation terms were consistently significant (i.e. in the pooled data as well as in the individual studies).

In order to investigate whether sleep disturbances are also associated with pre‐sleep worrying the following evening, we conducted a further exploratory analysis in this regard.

## 3 RESULTS

### 3.1 Descriptive statistics and associations between the variables

**FIGURE 2:**
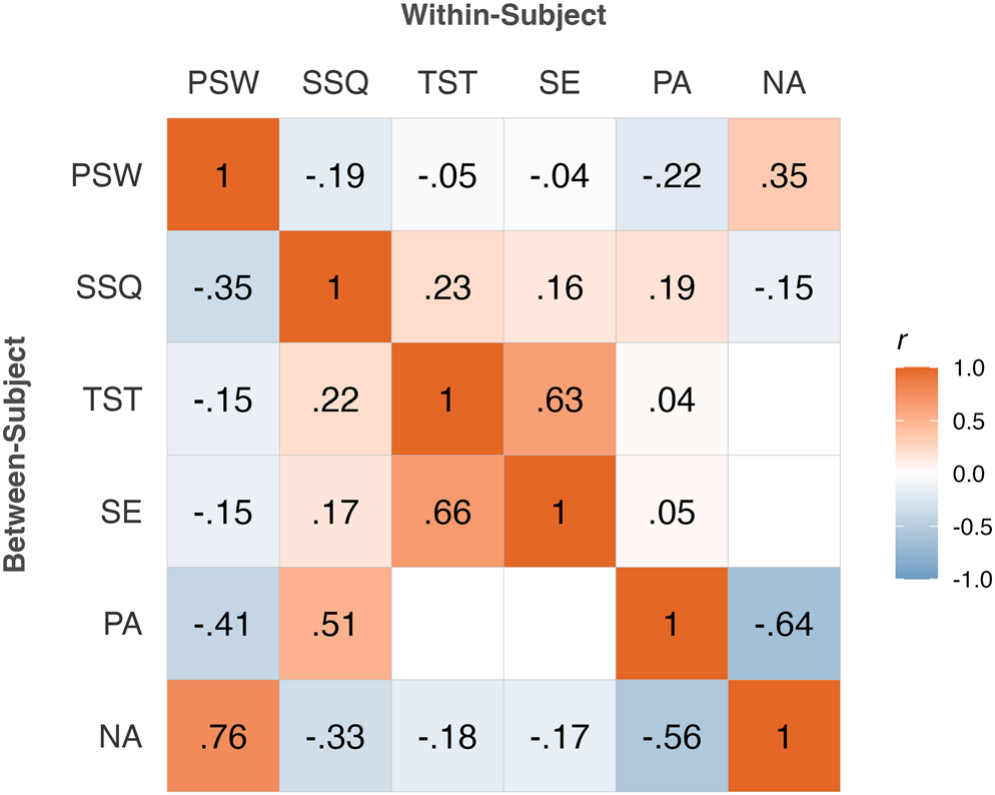
Within‐ and between‐subject Pearson correlations. NA, negative affect; PA, positive affect; PSW, pre‐sleep worrying; SE, sleep efficiency; SSQ, subjective sleep quality; TST, total sleep time. The lower left triangle displays between‐subject correlations, and the upper right triangle displays within‐subject correlations. Only significant correlations are displayed.

The demographic characteristics and descriptive statistics of the baseline questionnaires and ESM surveys are shown in Table 1. Although a non‐clinical sample was investigated, approximately 56% could have been categorized as poor sleepers. With regard to depressive symptoms, 33% of the sample showed moderate to severe symptoms, while the remaining 67% reported no to mild symptoms. Within‐ and between‐subject correlations of the variables are displayed in Figure 2. All correlations are in the expected direction, and most of the correlations are relatively similar for between‐ and within‐subject level.

### 3.2 Mediating role of subjective sleep quality for the association between pre‐sleep worrying and affective wellbeing (RQ1)

#### 3.2.1 Within‐subject analyses

In the within‐subject mediator model, more pre‐sleep worrying was associated with lower subjective sleep quality of the same night (*β* = −0.18, *p* < 0.001). In the within‐subject outcome model for positive affect, more pre‐sleep worrying was associated with lower positive affect on the subsequent day (*β* = −0.16, *p* < 0.001) and greater sleep disturbance (i.e. lower subjective sleep quality) was associated with lower positive affect (*β* = 0.14, *p* < 0.001). The mediation analysis showed a significant indirect effect (ACME; *β* = −0.02, *p* < 0.001), direct effect (ADE; *β* = −0.16, *p* < 0.001) and total effect (*β* = −0.19, *p* < 0.001), with a proportion of 12% of the total effect being mediated by subjective sleep quality.

In the within‐subject outcome model for negative affect, more pre‐sleep worrying was associated with higher negative affect on the subsequent day (*β* = 0.31, *p* < 0.001), and higher subjective sleep quality was associated with lower negative affect (*β* = −0.07, *p* < 0.001). The mediation analysis showed a significant indirect effect (ACME; *β* = 0.01, *p* < 0.001), direct effect (ADE; *β* = 0.31, *p* < 0.001) and total effect (*β* = 0.32, *p* < 0.001), with a proportion of 4% of the total effect being mediated by subjective sleep quality.

#### 3.2.2 Between‐subject analyses

In the between‐subject mediator model, more pre‐sleep worrying was associated with lower subjective sleep quality (*β* = −0.30, *p* < 0.001). In the between‐subject outcome model for positive affect, more pre‐sleep worrying was associated with lower positive affect (*β* = −0.26, *p* < 0.01), and greater sleep disturbance (i.e. lower subjective sleep quality) was associated with lower positive affect (*β* = 0.44, *p* < 0.001). Accordingly, the mediation analysis showed a significant indirect effect (ACME; *β* = −0.14, *p* < 0.001), direct effect (ADE; *β* = −0.27, *p* < 0.001) and total effect (*β* = −0.41, *p* < 0.001), with a proportion of 33% of the total effect being mediated by subjective sleep quality.

In the between‐subject outcome model for negative affect, more pre‐sleep worrying was associated with higher negative affect (*β* = 0.73, *p* < 0.001), but subjective sleep quality was not (*β* = −0.08, *p* = 0.185), which indicated that subjective sleep quality did not mediate the association between pre‐sleep worrying and negative affect.

### 3.3 Mediating role of objective SE and TST for the association between pre‐sleep worrying and affective wellbeing (RQ2)

#### 3.3.1 Within‐subject analyses

In the within‐subject mediator models, more pre‐sleep worrying was not associated with objective SE (*β* = 0.00, *p* = 0.989) or TST (*β* = −0.01, *p* = 0.886) of the same night. Also, SE and TST did not predict affective states on the subsequent day. Overall, these findings indicated that SE and TST did not mediate the association between pre‐sleep worrying and affective states on the within‐subject level. All results can be found in Tables S4 and S7.

#### 3.3.2 Between‐subject analyses

In the between‐subject mediator model, more pre‐sleep worrying was not associated with objective SE (*β* = −0.11, *p* = 0.121) or TST (*β* = −0.01, *p* = 0.989). Also, SE and TST were not associated with affective states. Overall, these findings indicated that SE and TST did not mediate the association between pre‐sleep worrying and affective states on the between‐subject level.

#### 3.3.3 Moderator analyses (RQ3)

Neither depressive symptoms nor sleep disturbances at baseline moderated the associations between pre‐sleep worrying, subjective sleep quality and affective states. Accordingly, no mediation analyses contrasting different value ranges of the moderators (–1 SD versus +1 SD) were calculated. All results of the moderation analyses are displayed in Tables S2, S3, S5, S6, S8 and S9.

### 3.4 Sleep disturbances and pre‐sleep worrying on the subsequent evening

Neither subjective sleep quality (*β* = −0.01, *p* = 0.698) nor SE (*β* = −0.02, *p* = 0.360) or TST (*β* = 0.00, *p* = 0.832) predicted pre‐sleep worrying the following night.

## 4 DISCUSSION

The results of this study show that pre‐sleep worrying is associated with decreased subjective sleep quality, as well as with decreased positive and increased negative affect on the following day. Subjective sleep quality also mediates the relationship between pre‐sleep worrying and positive as well as negative affect on the following day. Moreover, and importantly, objective SE and TST as measured by actigraphy do not mediate the association between pre‐sleep worrying and positive or negative affect on the following day. Further, pre‐sleep worrying is not associated with objective sleep parameters the following night. While subjective sleep quality is a partial mediator of the association between pre‐sleep worrying and positive as well as negative affect the following day, less than a third of the total effect of pre‐sleep worrying on positive affect is mediated. In the case of negative affect as outcome variable, only 4% are mediated. The lack of mediation by objective sleep parameters and only partial mediation by subjective sleep parameters suggests that the association between pre‐sleep worrying and the next day's affective wellbeing may be to a large degree reflective of a rather direct effect of pre‐sleep worrying on the levels of positive and negative affect the next day. Kalmbach et al. (2021) also suggest that cognitive‐emotional dysregulation (i.e. cognitive arousal and emotional states including mood and depression) is partly mediated by sleep disturbances/insomnia, calling this the depressogenic cycle of insomnia and cognitive arousal.

Our findings also reveal that the between‐subject associations in terms of direction and statistical significance are mostly similar to within‐subject associations, while the effect‐sizes were often substantially larger at the between‐subject level. Moreover, baseline levels of depressive and insomnia symptoms did not moderate the associations between pre‐sleep worrying, sleep indices and affective states. This indicates a relative uniformity of the observed relationships and of the mediation effect regardless of baseline levels of depressive or insomnia symptoms in this non‐clinical sample.

The findings are in line with the cognitive model of insomnia (Harvey, 2002), in that evening worries (e.g. about not being able to fall asleep) are associated with emotional arousal during the night and the next day, and could lead to decreased affective wellbeing. Furthermore, our results are consistent with previous studies showing associations between subjective sleep quality and increased positive and decreased negative affect the following day (Das‐Friebel et al., 2020; Hachenberger et al., 2023; Konjarski et al., 2018; Lenneis et al., 2024). Our finding of only modest or insignificant associations of actigraphy SE and TST with positive and negative affect on the following day are also consistent with past research that showed no associations of actigraphy SE and TST with positive and negative affect (Das‐Friebel et al., 2020; Konjarski et al., 2018; Lenneis et al., 2024).

The results of this study further indicate that pre‐sleep worrying is predictive of subjective sleep quality in the subsequent night. This is consistent with existing diary studies that studied subjective sleep quality (McGowan et al., 2016). However, objective SE and TST were not associated with pre‐sleep worrying in the subsequent evening. In line with this research is the result that the association of nocturnal worrying with objective SE is considerably smaller than the association with subjective sleep quality (van Laethem et al., 2016). Relatedly, the lack of mediation of the link between pre‐sleep worrying and affective wellbeing the next day by objective sleep parameters suggests that the putative role of pre‐sleep worrying for affective wellbeing is rather due to the subjective experience of sleep than objective sleep duration or efficiency as assessed by actigraphy. This is in line with the notion that how individuals perceive their sleep is more critical for affective wellbeing than the actual sleep metrics, at least when captured by actigraphy devices. Also, cross‐sectional research indicated that the variance in subjective sleep quality, which is explained by objective sleep measures including SE, is rather small (Kaplan et al., 2017).

The results suggest that interventions to improve sleep could also improve affective wellbeing due to the close link between sleep quality and positive as well as negative affect. In this line, CBT‐I has been shown to improve insomnia severity, as well as comorbid mental health symptoms including depressive and anxiety symptoms (Ballesio et al., 2021; Hertenstein et al., 2022; Perrault et al., 2022). In addition, CBT‐I has positive effects on worrying, with greater effects on sleep‐related measures of repetitive negative thinking than on general measures of cognitive arousal (Ballesio et al., 2021). In line with that, also mindfulness‐based therapy for insomnia decreases maladaptive sleep‐related cognitions (Ong et al., 2018). Moreover, the results also suggest that psychological interventions to reduce worrying may also be beneficial for sleep (McCarrick et al., 2021).

Even if the present study does not allow any causal conclusions to be drawn, we consider the intensive longitudinal design a strength of the study allowing consideration of the temporal order of effects, which is assumed to improve our understanding of within‐person mechanisms. Comparisons of effect‐sizes within the two samples also show that results are fairly similar indicating robustness of the findings. To the best of our knowledge, this study is also the first to investigate the *mediating* role of subjective and objectively measured sleep quality regarding the relationship between pre‐sleep worrying and positive and negative affect on the following day using an intensive longitudinal design allowing separate analyses of within‐subject and between‐subject variance.

However, the current study also has several limitations that should not be ignored. First, our sample mainly consisted of women (83%; similar in Narmandakh et al., 2021). Therefore, generalization of results for all genders is difficult. Second, the results may not be generalized to clinical or subclinical samples, although the number of individuals with increased levels of sleep disturbances was relatively high in our study. Future studies will have to investigate the associations between sleep, worrying and affect in insomnia samples. Third, our study did not measure sleep‐related worrying specifically for which CBT‐I shows stronger effects than for general worrying (Ballesio et al., 2021). As a consequence, future intensive longitudinal studies should investigate sleep‐related worrying and its associations with sleep and affect. Fourth, also other directions of effects are conceivable, including, for example, that lying awake at night due to sleep problems gives more opportunities to worry and ruminate, which in turn may have an effect on the affect the next day (Lovato & Gradisar, 2014). Likewise, a vicious circle may emerge, as for instance low levels of positive affect during the day might trigger night‐time worries and sleep problems (Ong et al., 2018). However, studies exist that were not able to show a prediction of sleep quality by positive and negative affect (Bouwmans et al., 2017). This suggests that the association of sleep with affective wellbeing on the following day may be stronger than the association of affective wellbeing with sleep in the following night. Furthermore, our exploratory analyses show that sleep at night was not related to pre‐sleep worrying the following evening. Further research should also include other age groups when looking at the interaction between sleep, worries and affect. Fifth, the ESM variables for pre‐sleep worrying and subjective sleep quality were each derived from a single item. While single‐item measures are common in ambulatory assessment studies due to their ease of use and low burden for participants, their validity should be further investigated in future research (Allen et al., 2022). Sixth, pre‐sleep worrying and subjective sleep quality were assessed at the same time (i.e. in the morning after), which may have introduced bias. Finally, our objective measures of SE and TST have been derived from actigraphy. While actigraphy has confirmed validity in measuring SE and TST compared with polysomnography, it does not have a high accuracy in detecting wakefulness at night (Marino et al., 2013). It is therefore possible that the use of polysomnography or other recently developed methods to derive detailed hypnograms (Topalidis et al., 2023) would have allowed to find significant effects regarding objective SE and TST. Relatedly it is possible that other objective indicators of sleep quality such as rapid eye movement (REM)‐sleep instability or the number of sleep stage transitions (Laffan et al., 2010; Riemann et al., 2024), which can only be derived from polysomnography, would be more strongly associated with affective wellbeing the next day.

Addressing pre‐sleep worrying and improving subjective sleep quality could be potential pathways to enhance affective wellbeing and promoting better mental health in young adults. However, sleep as measured by actigraphy appears not to be relevant for the link between pre‐sleep worrying and affective wellbeing the following day.

## AUTHOR CONTRIBUTIONS

**Anika Werner:** Writing – review and editing; writing – original draft; conceptualization; methodology; investigation. **Justin Hachenberger:** Writing – review and editing; conceptualization; investigation; writing – original draft; methodology; formal analysis; data curation. **Kai Spiegelhalder:** Writing – review and editing; conceptualization. **Jana‐Elisa Rueth:** Writing – review and editing. **Angelika A. Schlarb:** Writing – review and editing. **Arnold Lohaus:** Writing – review and editing. **Sakari Lemola:** Writing – review and editing; supervision; conceptualization; investigation; writing – original draft; methodology; project administration.

## FUNDING INFORMATION

This research received no external funding.

## CONFLICT OF INTEREST STATEMENT

All authors have no known conflict of interest to disclose.

## Supporting information


**DATA S1** Supporting Information.

## Data Availability

The data that support the findings of this study are available on request from the corresponding author. The data are not publicly available due to privacy or ethical restrictions.
