# Supplementary material for: Subjective sleep quality, but not objective sleep measures, mediates the relationship between pre‐sleep worrying and affective wellbeing
Source: J Sleep Res. 2025 Jan 30;34(6):e14467. doi: 10.1111/jsr.14467 (PMC12592825; doi:10.1111/jsr.14467)
Supplement: Supplementary file 1 — DATA S1 Supporting Information. [file JSR-34-e14467-s001.pdf]

Subjective Sleep Quality, but not Objective Sleep Measures, Mediates the Relationship Between Pre-Sleep Worrying and Affective Wellbeing

Supplementary Material

Anika Werner, Justin Hachenberger, Kai Spiegelhalder, Jana-Elisa Rüth, Angelika A. Schlarb, Arnold Lohaus, Sakari Lemola

Corresponding author:

Dr. Anika Werner
Bielefeld University
Faculty of Psychology and Sports Science, Department of Psychology
Developmental Psychology and Developmental Psychopathology
P.O. Box 10 01 31
D-33501 Bielefeld
Anika.werner@uni-bielefeld.de

Supplementary Table 1

Results of the mediator model, outcome model, and subsequent mediation analysis for subjective sleep quality (SSQ) as the mediator

			Within-Subject							Between-Subject						
			Pooled Data			Study 1		Study 2		Pooled Data			Study 1		Study 2	
Outcome	Model	Predictor	Est.	*CI*	*p*	Est.	*p*	Est.	*p*	Est.	*CI*	*p*	Est.	*p*	Est.	*p*
PA	Path a (SSQ)	Worries	–0.18	[–0.21, –0.15]	**< .001**	–0.19	**< .001**	–0.17	**< .001**	–0.30	[–0.44, –0.17]	**< .001**	–0.27	**< .01**	–0.38	**< .01**
	Path b + c	Worries	–0.16	[–0.20, –0.13]	**< .001**	–0.14	**< .001**	–0.19	**< .001**	–0.26	[–0.38, –0.14]	**< .001**	–0.30	**< .001**	–0.24	.063
		SSQ	0.14	[0.10, 0.17]	**< .001**	0.10	**< .001**	0.18	**< .001**	0.44	[0.32, 0.56]	**< .001**	0.44	**< .001**	0.43	**< .001**
	Mediation	ACME	–0.02	[–0.03, –0.01]	**< .001**	–0.02	**< .001**	–0.03	**< .001**	–0.14	[–0.20, –0.08]	**< .001**	–0.13	**< .001**	–0.17	**< .001**
		ADE	–0.16	[–0.21, –0.13]	**< .001**	–0.14	**< .001**	–0.19	**< .001**	–0.27	[–0.38, –0.17]	**< .001**	–0.30	**< .001**	–0.23	**< .001**
		TE	–0.19	[–0.23, –0.15]	**< .001**	–0.16	**< .001**	–0.22	**< .001**	–0.41	[–0.53, –0.30]	**< .001**	–0.43	**< .001**	–0.41	**< .001**
		Prop. Mediated	0.12	[0.07, 0.19]	**< .001**	0.11	**< .001**	0.14	**< .001**	0.33	[0.20, 0.49]	**< .001**	0.30	**< .001**	0.41	**< .001**
NA	Path a (SSQ)	Worries	–0.18	[–0.21, –0.15]	**< .001**	–0.19	**< .001**	–0.17	**< .001**	–0.30	[–0.44, –0.17]	**< .001**	–0.27	**< .01**	–0.38	**< .01**
	Path b + c	Worries	0.31	[0.27, 0.34]	**< .001**	0.25	**< .001**	0.38	**< .001**	0.73	[0.63, 0.83]	**< .001**	0.70	**< .001**	0.80	**< .001**
		SSQ	–0.07	[–0.10, –0.03]	**< .001**	–0.06	**< .05**	–0.09	**< .01**	–0.08	[–0.18, 0.01]	.185	–0.15	**< .05**	0.01	.962
	Mediation	ACME	0.01	[0.01, 0.02]	**< .001**	0.01	.090	0.02	**< .001**	0.02	[–0.01, 0.05]	.158	0.04	**< .001**	0.00	.988
		ADE	0.31	[0.26, 0.35]	**< .001**	0.25	**< .001**	0.39	**< .001**	0.74	[0.65, 0.83]	**< .001**	0.71	**< .001**	0.78	**< .001**
		TE	0.32	[0.28, 0.36]	**< .001**	0.26	**< .001**	0.40	**< .001**	0.77	[0.68, 0.86]	**< .001**	0.76	**< .001**	0.78	**< .001**
		Prop. Mediated	0.04	[0.02, 0.07]	**< .001**	0.04	.090	0.04	**< .001**	0.03	[–0.01, 0.07]	.158	0.05	**< .001**	0.00	.988

Note. SSQ subjective sleep quality; ACME average causal mediation effect (indirect effect); ADE average direct effect; TE total effect; Prop. Mediated proportion mediated. Please note that the mediator models for PA and NA are identical, but are displayed twice above each outcome model for reasons of overview. The estimates for the covariates are not displayed.

Supplementary Table 2

Results of the mediator model, outcome model, and subsequent mediation analysis for subjective sleep quality (SSQ) as the mediator and insomnia symptoms (IS) as a moderator

			Within-Subject							Between-Subject						
			Pooled Data			Study 1		Study 2		Pooled Data			Study 1		Study 2	
Outcome	Model	Predictor	Est.	*CI*	*p*	Est.	*p*	Est.	*p*	Est.	*CI*	*p*	Est.	*p*	Est.	*p*
PA	Path a (SSQ)	Worries	–0.18	[–0.22, –0.15]	**< .001**	–0.19	**< .001**	–0.17	**< .001**	–0.21	[–0.33, –0.08]	**< .01**	–0.13	.268	–0.25	**< .05**
		Worries x IS	–0.01	[–0.04, 0.03]	.871	–0.01	.845	–0.01	.933	0.08	[–0.02, 0.19]	.238	0.01	.920	0.18	.141
	Path b + c	Worries	–0.16	[–0.20, –0.13]	**< .001**	–0.15	**< .001**	–0.19	**< .001**	–0.28	[–0.40, –0.15]	**< .001**	–0.32	**< .001**	–0.23	.070
		SSQ	0.13	[0.10, 0.17]	**< .001**	0.10	**< .001**	0.18	**< .001**	0.49	[0.36, 0.62]	**< .001**	0.52	**< .001**	0.41	**< .001**
		Worries x IS	0.01	[–0.03, 0.05]	.920	0.00	.943	0.02	.669	0.01	[–0.11, 0.12]	.989	–0.07	.527	0.18	.188
		SSQ x IS	0.00	[–0.04, 0.04]	.989	–0.02	.712	0.02	.638	0.01	[–0.11, 0.13]	.989	0.01	.959	–0.02	.951
NA	Path a (SSQ)	Worries	–0.18	[–0.22, –0.15]	**< .001**	–0.19	**< .001**	–0.17	**< .001**	–0.21	[–0.33, –0.08]	**< .01**	–0.13	.268	–0.25	**< .05**
		Worries x IS	–0.01	[–0.04, 0.03]	.871	–0.01	.845	–0.01	.933	0.08	[–0.02, 0.19]	.238	0.01	.920	0.18	.141
	Path b + c	Worries	0.31	[0.27, 0.34]	**< .001**	0.26	**< .001**	0.38	**< .001**	0.74	[0.64, 0.84]	**< .001**	0.73	**< .001**	0.78	**< .001**
		SSQ	–0.07	[–0.10, –0.04]	**< .001**	–0.06	**< .05**	–0.09	**< .01**	–0.08	[–0.19, 0.02]	.231	–0.19	**< .05**	0.08	.577
		Worries x IS	–0.01	[–0.05, 0.03]	.853	0.02	.684	–0.05	.182	–0.08	[–0.17, 0.02]	.196	–0.06	.527	–0.17	.072
		SSQ x IS	–0.03	[–0.07, 0.00]	.132	–0.01	.783	–0.06	.097	–0.03	[–0.13, 0.07]	.819	–0.05	.653	–0.04	.880

Note. SSQ subjective sleep quality; ISS insomnia symptom severity; ACME average causal mediation effect (indirect effect); ADE average direct effect; TE total effect; Prop. Mediated proportion mediated. Please note that the mediator models for PA and NA are identical, but are displayed twice above each outcome model for reasons of overview. The estimates for the covariates are not displayed.

Supplementary Table 3

Results of the mediator model, outcome model, and subsequent mediation analysis for subjective sleep quality (SSQ) as the mediator and depressive symptoms (PHQ-9) as a moderator

			Within-Subject							Between-Subject						
			Pooled Data			Study 1		Study 2		Pooled Data			Study 1		Study 2	
Outcome	Model	Predictor	Est.	*CI*	*p*	Est.	*p*	Est.	*p*	Est.	*CI*	*p*	Est.	*p*	Est.	*p*
PA	Path a (SSQ)	Worries	–0.18	[–0.21, –0.15]	**< .001**	–0.19	**< .001**	–0.17	**< .001**	–0.18	[–0.32, –0.04]	**< .05**	–0.13	.331	–0.22	.153
		Worries x PHQ9	0.00	[–0.04, 0.03]	.989	–0.02	.600	0.02	.617	0.05	[–0.07, 0.17]	.644	–0.02	.918	0.14	.283
	Path b + c	Worries	–0.16	[–0.20, –0.13]	**< .001**	–0.15	**< .001**	–0.19	**< .001**	–0.20	[–0.33, –0.08]	**< .01**	–0.27	**< .01**	–0.10	.630
		SSQ	0.14	[0.10, 0.17]	**< .001**	0.10	**< .001**	0.18	**< .001**	0.38	[0.25, 0.50]	**< .001**	0.37	**< .001**	0.35	**< .01**
		Worries x PHQ9	0.00	[–0.04, 0.04]	.990	0.01	.889	–0.01	.927	0.01	[–0.11, 0.12]	.989	–0.10	.388	0.06	.780
		SSQ x PHQ9	–0.01	[–0.04, 0.03]	.945	–0.03	.333	0.03	.418	–0.03	[–0.16, 0.09]	.861	–0.14	.203	0.03	.951
NA	Path a (SSQ)	Worries	–0.18	[–0.21, –0.15]	**< .001**	–0.19	**< .001**	–0.17	**< .001**	–0.18	[–0.32, –0.04]	**< .05**	–0.13	.331	–0.22	.153
		Worries x PHQ9	0.00	[–0.04, 0.03]	.989	–0.02	.600	0.02	.617	0.05	[–0.07, 0.17]	.644	–0.02	.918	0.14	.283
	Path b + c	Worries	0.31	[0.27, 0.34]	**< .001**	0.25	**< .001**	0.38	**< .001**	0.65	[0.55, 0.75]	**< .001**	0.64	**< .001**	0.64	**< .001**
		SSQ	–0.07	[–0.10, –0.04]	**< .001**	–0.06	**< .05**	–0.09	**< .01**	–0.02	[–0.12, 0.08]	.942	–0.12	.177	0.08	.480
		Worries x PHQ9	0.00	[–0.04, 0.03]	.989	0.00	.975	–0.01	.933	0.01	[–0.09, 0.10]	.989	–0.04	.789	0.04	.822
		SSQ x PHQ9	–0.03	[–0.06, 0.01]	.268	–0.01	.794	–0.05	.218	–0.01	[–0.11, 0.09]	.989	–0.07	.558	0.00	1.000

Note. SSQ subjective sleep quality; PHQ9 Patient Health Questionnaire 9; ACME average causal mediation effect (indirect effect); ADE average direct effect; TE total effect; Prop. Mediated proportion mediated. Please note that the mediator models for PA and NA are identical, but are displayed twice above each outcome model for reasons of overview. The estimates for the covariates are not displayed.

Supplementary Table 4

Results of the mediator model, outcome model, and subsequent mediation analysis for sleep efficiency (Acc-SE) as the mediator

			Within-Subject							Between-Subject						
			Pooled Data			Study 1		Study 2		Pooled Data			Study 1		Study 2	
Outcome	Model	Predictor	Est.	*CI*	*p*	Est.	*p*	Est.	*p*	Est.	*CI*	*p*	Est.	*p*	Est.	*p*
PA	Path a (Acc-SE)	Worries	0.00	[–0.03, 0.03]	.989	0.00	.918	0.01	.927	–0.11	[–0.22, 0.00]	.121	–0.10	.275	–0.11	.467
	Path b + c	Worries	–0.16	[–0.19, –0.12]	**< .001**	–0.14	**< .001**	–0.19	**< .001**	–0.26	[–0.38, –0.14]	**< .001**	–0.30	**< .001**	–0.24	.063
		Acc-SE	0.01	[–0.03, 0.05]	.890	–0.02	.547	0.05	.163	0.02	[–0.13, 0.16]	.989	0.02	.941	0.07	.860
	Mediation	ACME	0.00	[0.00, 0.00]	.989	0.00	.976	0.00	.951	0.00	[–0.02, 0.02]	.989	0.00	.959	–0.01	.927
		ADE	–0.16	[–0.20, –0.12]	**< .001**	–0.14	**< .001**	–0.19	**< .001**	–0.26	[–0.38, –0.15]	**< .001**	–0.29	**< .001**	–0.25	**< .05**
		TE	–0.16	[–0.20, –0.12]	**< .001**	–0.14	**< .001**	–0.19	**< .001**	–0.26	[–0.38, –0.15]	**< .001**	–0.29	**< .001**	–0.26	**< .001**
		Prop. Mediated	0.00	[0.00, 0.00]	.989	0.00	.976	0.00	.951	0.01	[–0.08, 0.11]	.989	0.00	.959	0.02	.927
NA	Path a (Acc-SE)	Worries	0.00	[–0.03, 0.03]	.989	0.00	.918	0.01	.927	–0.11	[–0.22, 0.00]	.121	–0.10	.275	–0.11	.467
	Path b + c	Worries	0.31	[0.27, 0.34]	**< .001**	0.25	**< .001**	0.38	**< .001**	0.73	[0.63, 0.83]	**< .001**	0.70	**< .001**	0.80	**< .001**
		Acc-SE	0.01	[–0.03, 0.04]	.890	0.03	.433	–0.02	.778	0.05	[–0.07, 0.17]	.614	0.18	.09	–0.10	.495
	Mediation	ACME	0.00	[0.00, 0.00]	.989	0.00	.916	0.00	.973	–0.01	[–0.02, 0.01]	.644	–0.02	.238	0.01	.597
		ADE	0.31	[0.27, 0.35]	**< .001**	0.25	**< .001**	0.39	**< .001**	0.73	[0.64, 0.86]	**< .001**	0.70	**< .001**	0.79	**< .001**
		TE	0.31	[0.27, 0.35]	**< .001**	0.25	**< .001**	0.39	**< .001**	0.73	[0.63, 0.86]	**< .001**	0.69	**< .001**	0.80	**< .001**
		Prop. Mediated	0.00	[0.00, 0.00]	.989	0.00	.916	0.00	.973	–0.01	[–0.03, 0.01]	.644	–0.02	.238	0.01	.597

Note. Acc-SE objective sleep efficiency; ACME average causal mediation effect (indirect effect); ADE average direct effect; TE total effect; Prop. Mediated proportion mediated. Please note that the mediator models for PA and NA are identical, but are displayed twice above each outcome model for reasons of overview. The estimates for the covariates are not displayed.

Supplementary Table 5

Results of the mediator model, outcome model, and subsequent mediation analysis for sleep efficiency (Acc-SE) as the mediator and insomnia symptoms (IS) as a moderator

			Within-Subject							Between-Subject						
			Pooled Data			Study 1		Study 2		Pooled Data			Study 1		Study 2	
Outcome	Model	Predictor	Est.	*CI*	*p*	Est.	*p*	Est.	*p*	Est.	*CI*	*p*	Est.	*p*	Est.	*p*
PA	Path a (Acc-SE)	Worries	0.00	[–0.03, 0.03]	.990	–0.01	.882	0.01	.927	–0.08	[–0.2, 0.03]	.267	–0.06	.597	–0.11	.420
		Worries x IS	–0.01	[–0.04, 0.02]	.625	–0.03	.293	0.00	.951	–0.09	[–0.18, 0.00]	.123	–0.05	.558	–0.16	.133
	Path b + c	Worries	–0.16	[–0.2, –0.12]	**< .001**	–0.14	**< .001**	–0.19	**< .001**	–0.26	[–0.39, –0.14]	**< .001**	–0.31	**< .001**	–0.19	.152
		Acc-SE	0.01	[–0.03, 0.05]	.849	–0.02	.577	0.06	.149	0.00	[–0.15, 0.15]	.990	0.00	.987	0.09	.722
		Worries x IS	0.01	[–0.03, 0.05]	.823	0.00	.971	0.02	.701	0.02	[–0.08, 0.12]	.942	–0.06	.487	0.22	.068
		Acc-SE x IS	0.02	[–0.01, 0.05]	.289	0.01	.710	0.03	.371	0.11	[0.01, 0.22]	.082	0.07	.554	0.17	.077
NA	Path a (Acc-SE)	Worries	0.00	[–0.03, 0.03]	.990	–0.01	.882	0.01	.927	–0.08	[–0.20, 0.03]	.267	–0.06	.597	–0.11	.420
		Worries x IS	–0.01	[–0.04, 0.02]	.625	–0.03	.293	0.00	.951	–0.09	[–0.18, 0.00]	.123	–0.05	.558	–0.16	.133
	Path b + c	Worries	0.31	[0.27, 0.34]	**< .001**	0.26	**< .001**	0.38	**< .001**	0.74	[0.64, 0.84]	**< .001**	0.73	**< .001**	0.77	**< .001**
		Acc-SE	0.01	[–0.03, 0.04]	.942	0.03	.438	–0.02	.774	0.04	[–0.09, 0.16]	.855	0.13	.309	–0.14	.254
		Worries x IS	0.00	[–0.04, 0.03]	.989	0.02	.540	–0.04	.324	–0.06	[–0.15, 0.02]	.256	–0.03	.792	–0.16	.072
		Acc-SE x IS	0.00	[–0.03, 0.03]	.990	0.02	.625	–0.02	.692	0.01	[–0.08, 0.10]	.989	0.05	.633	–0.02	.927

Note. Acc-SE objective sleep efficiency; ISS insomnia symptom severity; ACME average causal mediation effect (indirect effect); ADE average direct effect; TE total effect; Prop. Mediated proportion mediated. Please note that the mediator models for PA and NA are identical, but are displayed twice above each outcome model for reasons of overview. The estimates for the covariates are not displayed.

Supplementary Table 6

Results of the mediator model, outcome model, and subsequent mediation analysis for sleep efficiency (Acc-SE) as the mediator and depressive symptoms (PHQ-9) as a moderator

			Within-Subject							Between-Subject						
			Pooled Data			Study 1		Study 2		Pooled Data			Study 1		Study 2	
Outcome	Model	Predictor	Est.	*CI*	*p*	Est.	*p*	Est.	*p*	Est.	*CI*	*p*	Est.	*p*	Est.	*p*
PA	Path a (Acc-SE)	Worries	0.00	[–0.03, 0.03]	.990	–0.01	.889	0.01	.927	–0.12	[–0.24, 0.00]	.087	–0.13	.166	–0.10	.597
		Worries x PHQ9	–0.03	[–0.06, 0.00]	.082	–0.04	.090	–0.02	.687	–0.06	[–0.16, 0.03]	.356	–0.07	.454	–0.02	.951
	Path b + c	Worries	–0.16	[–0.19, –0.12]	**< .001**	–0.14	**< .001**	–0.19	**< .001**	–0.19	[–0.32, –0.06]	**< .01**	–0.26	**< .01**	–0.09	.684
		Acc-SE	0.01	[–0.03, 0.05]	.888	–0.02	.569	0.05	.164	0.05	[–0.10, 0.19]	.819	0.03	.889	0.10	.659
		Worries x PHQ9	0.00	[–0.03, 0.04]	.989	0.02	.694	–0.01	.887	0.03	[–0.07, 0.14]	.825	–0.02	.889	0.07	.659
		Acc-SE x PHQ9	0.02	[–0.02, 0.05]	.530	0.03	.409	0.01	.951	0.10	[–0.03, 0.22]	.263	0.02	.916	0.20	.126
NA	Path a (Acc-SE)	Worries	0.00	[–0.03, 0.03]	.990	–0.01	.889	0.01	.927	–0.12	[–0.24, 0.00]	.087	–0.13	.166	–0.10	.597
		Worries x PHQ9	–0.03	[–0.06, 0.00]	.082	–0.04	.090	–0.02	.687	–0.06	[–0.16, 0.03]	.356	–0.07	.454	–0.02	.951
	Path b + c	Worries	0.31	[0.27, 0.34]	**< .001**	0.25	**< .001**	0.38	**< .001**	0.65	[0.55, 0.75]	**< .001**	0.65	**< .001**	0.63	**< .001**
		Acc-SE	0.01	[–0.03, 0.04]	.893	0.03	.437	–0.02	.778	0.03	[–0.09, 0.15]	.886	0.14	.192	–0.12	.328
		Worries x PHQ9	0.00	[–0.04, 0.04]	.990	0.00	.998	0.00	.958	0.01	[–0.07, 0.09]	.989	0.01	.975	0.03	.917
		Acc-SE x PHQ9	–0.01	[–0.04, 0.02]	.890	–0.01	.792	0.00	.951	–0.01	[–0.11, 0.09]	.989	0.07	.503	–0.11	.296

Note. Acc-SE objective sleep efficiency; PHQ9 Patient Health Questionnaire 9; ACME average causal mediation effect (indirect effect); ADE average direct effect; TE total effect; Prop. Mediated proportion mediated. Please note that the mediator models for PA and NA are identical, but are displayed twice above each outcome model for reasons of overview. The estimates for the covariates are not displayed.

Supplementary Table 7

Results of the mediator model, outcome model, and subsequent mediation analysis for total sleep time (Acc-TST) as the mediator

			Within-Subject							Between-Subject						
			Pooled Data			Study 1		Study 2		Pooled Data			Study 1		Study 2	
Outcome	Model	Predictor	Est.	*CI*	*p*	Est.	*p*	Est.	*p*	Est.	*CI*	*p*	Est.	*p*	Est.	*p*
PA	Path a (Acc-TST)	Worries	–0.01	[–0.04, 0.02]	.886	–0.01	.834	–0.01	.951	–0.01	[–0.12, 0.10]	.989	–0.06	.569	0.03	.927
	Path b + c	Worries	–0.16	[–0.20, –0.12]	**< .001**	–0.14	**< .001**	–0.19	**< .001**	–0.26	[–0.38, –0.14]	**< .001**	–0.30	**< .001**	–0.24	.063
		Acc-TST	0.00	[–0.04, 0.04]	.989	0.03	.487	–0.04	.462	–0.11	[–0.26, 0.04]	.267	–0.08	.586	–0.19	.281
	Mediation	ACME	0.00	[0.00, 0.00]	.989	0.00	.918	0.00	.953	0.00	[–0.01, 0.02]	.921	0.00	.916	–0.01	.953
		ADE	–0.16	[–0.2, –0.13]	**< .001**	–0.14	**< .001**	–0.19	**< .001**	–0.27	[–0.36, –0.14]	**< .001**	–0.29	**< .001**	–0.24	**< .001**
		TE	–0.16	[–0.2, –0.13]	**< .001**	–0.14	**< .001**	–0.19	**< .001**	–0.26	[–0.36, –0.13]	**< .001**	–0.29	**< .001**	–0.25	**< .001**
		Prop. Mediated	0.00	[–0.01, 0.01]	.989	0.00	.918	0.00	.953	–0.01	[–0.12, 0.05]	.921	0.00	.916	0.01	.953
NA	Path a (Acc-TST)	Worries	–0.01	[–0.04, 0.02]	.886	–0.01	.834	–0.01	.951	–0.01	[–0.12, 0.10]	.989	–0.06	.569	0.03	.927
	Path b + c	Worries	0.31	[0.27, 0.34]	**< .001**	0.25	**< .001**	0.38	**< .001**	0.73	[0.63, 0.83]	**< .001**	0.70	**< .001**	0.80	**< .001**
		Acc-TST	0.00	[–0.03, 0.04]	.989	–0.01	.734	0.03	.648	–0.05	[–0.17, 0.07]	.683	–0.12	.305	0.02	.953
	Mediation	ACME	0.00	[0.00, 0.00]	.942	0.00	.987	0.00	.962	0.00	[–0.01, 0.01]	.989	0.01	.600	0.00	1.000
		ADE	0.31	[0.27, 0.35]	**< .001**	0.25	**< .001**	0.38	**< .001**	0.72	[0.64, 0.83]	**< .001**	0.70	**< .001**	0.77	**< .001**
		TE	0.31	[0.27, 0.35]	**< .001**	0.25	**< .001**	0.38	**< .001**	0.72	[0.63, 0.84]	**< .001**	0.71	**< .001**	0.78	**< .001**
		Prop. Mediated	0.00	[0.00, 0.00]	.942	0.00	.987	0.00	.962	0.00	[–0.02, 0.01]	.989	0.01	.600	0.00	1.000

Note. Acc-SE objective sleep efficiency; ACME average causal mediation effect (indirect effect); ADE average direct effect; TE total effect; Prop. Mediated proportion mediated. Please note that the mediator models for PA and NA are identical, but are displayed twice above each outcome model for reasons of overview. The estimates for the covariates are not displayed.

Supplementary Table 8

Results of the mediator model, outcome model, and subsequent mediation analysis for total sleep time (Acc-TST) as the mediator and insomnia symptoms (IS) as a moderator

			Within-Subject							Between-Subject						
			Pooled Data			Study 1		Study 2		Pooled Data			Study 1		Study 2	
Outcome	Model	Predictor	Est.	*CI*	*p*	Est.	*p*	Est.	*p*	Est.	*CI*	*p*	Est.	*p*	Est.	*p*
PA	Path a (Acc-TST)	Worries	0.00	[–0.03, 0.02]	.945	–0.01	.889	–0.01	.951	–0.01	[–0.12, 0.11]	.989	–0.11	.265	0.06	.788
		Worries x IS	0.01	[–0.02, 0.04]	.614	–0.01	.712	0.04	.133	0.04	[–0.05, 0.13]	.613	0.07	.333	0.00	.999
	Path b + c	Worries	–0.16	[–0.2, –0.13]	**< .001**	–0.14	**< .001**	–0.19	**< .001**	–0.28	[–0.4, –0.15]	**< .001**	–0.32	**< .001**	–0.23	.076
		Acc-TST	0.00	[–0.04, 0.04]	.989	0.03	.496	–0.04	.451	–0.11	[–0.27, 0.05]	.297	–0.11	.527	–0.17	.389
		Worries x IS	0.01	[–0.03, 0.05]	.855	0.00	.98	0.02	.727	0.00	[–0.10, 0.10]	.989	–0.07	.399	0.19	.133
		Acc-TST x IS	0.00	[–0.03, 0.04]	.995	0.00	.920	0.01	.951	0.00	[–0.10, 0.10]	.994	–0.02	.902	0.02	.951
NA	Path a (Acc-TST)	Worries	0.00	[–0.03, 0.02]	.945	–0.01	.889	–0.01	.951	–0.01	[–0.12, 0.11]	.989	–0.11	.265	0.06	.788
		Worries x ISS	0.01	[–0.02, 0.04]	.614	–0.01	.712	0.04	.133	0.04	[–0.05, 0.13]	.613	0.07	.333	0.00	.999
	Path b + c	Worries	0.31	[0.27, 0.34]	**< .001**	0.26	**< .001**	0.38	**< .001**	0.74	[0.64, 0.85]	**< .001**	0.72	**< .001**	0.78	**< .001**
		Acc-TST	0.00	[–0.03, 0.04]	.989	–0.01	.766	0.03	.612	–0.07	[–0.20, 0.06]	.496	–0.16	.192	0.04	.898
		Worries x IS	0.00	[–0.04, 0.03]	.989	0.02	.586	–0.04	.352	–0.07	[–0.15, 0.02]	.227	–0.03	.717	–0.16	.077
		Acc-TST x IS	0.00	[–0.03, 0.03]	.989	0.00	.998	0.01	.951	0.05	[–0.03, 0.13]	.382	0.11	.127	0.00	1.000

Note. Acc-SE objective sleep efficiency; ISS insomnia symptom severity; ACME average causal mediation effect (indirect effect); ADE average direct effect; TE total effect; Prop. Mediated proportion mediated. Please note that the mediator models for PA and NA are identical, but are displayed twice above each outcome model for reasons of overview. The estimates for the covariates are not displayed.

Supplementary Table 9

Results of the mediator model, outcome model, and subsequent mediation analysis for total sleep time (Acc-TST) as the mediator and depressive symptoms (PHQ-9) as a moderator

			Within-Subject							Between-Subject						
			Pooled Data			Study 1		Study 2		Pooled Data			Study 1		Study 2	
Outcome	Model	Predictor	Est.	*CI*	*p*	Est.	*p*	Est.	*p*	Est.	*CI*	*p*	Est.	*p*	Est.	*p*
PA	Path a (Acc-TST)	Worries	–0.01	[–0.04, 0.02]	.890	–0.01	.847	–0.01	.951	0.00	[–0.11, 0.12]	.989	–0.09	.415	0.09	.671
		Worries x PHQ9	0.02	[–0.01, 0.05]	.235	0.02	.460	0.03	.514	0.06	[–0.04, 0.16]	.391	0.12	.107	–0.03	.927
	Path b + c	Worries	–0.16	[–0.20, –0.12]	**< .001**	–0.14	**< .001**	–0.19	**< .001**	–0.20	[–0.33, –0.07]	**< .01**	–0.26	**< .01**	–0.09	.671
		Acc-TST	0.00	[–0.04, 0.04]	.989	0.03	.496	–0.04	.467	–0.16	[–0.31, –0.01]	.084	–0.11	.482	–0.24	.133
		Worries x PHQ9	0.00	[–0.03, 0.04]	.989	0.01	.778	–0.02	.828	0.03	[–0.08, 0.13]	.882	–0.02	.888	0.06	.759
		Acc-TST x PHQ9	–0.01	[–0.04, 0.03]	.942	–0.01	.709	0.00	1.000	0.09	[–0.04, 0.22]	.283	0.10	.432	0.06	.852
NA	Path a (Acc-TST)	Worries	–0.01	[–0.04, 0.02]	.890	–0.01	.847	–0.01	.951	0.00	[–0.11, 0.12]	.989	–0.09	.415	0.09	.671
		Worries x PHQ9	0.02	[–0.01, 0.05]	.235	0.02	.460	0.03	.514	0.06	[–0.04, 0.16]	.391	0.12	.107	–0.03	.927
	Path b + c	Worries	0.31	[0.27, 0.34]	**< .001**	0.25	**< .001**	0.38	**< .001**	0.65	[0.55, 0.75]	**< .001**	0.64	**< .001**	0.63	**< .001**
		Acc-TST	0.00	[–0.03, 0.04]	.989	–0.01	.740	0.03	.658	–0.01	[–0.13, 0.11]	.989	–0.12	.358	0.08	.648
		Worries x PHQ9	0.00	[–0.04, 0.04]	.989	0.00	.976	0.00	1.000	0.01	[–0.08, 0.09]	.989	0.00	1.000	0.03	.887
		Acc-TST x PHQ9	0.00	[–0.03, 0.03]	.989	0.02	.569	–0.02	.722	–0.05	[–0.16, 0.05]	.524	–0.03	.872	–0.06	.696

Note. Acc-SE objective sleep efficiency; PHQ9 Patient Health Questionnaire 9; ACME average causal mediation effect (indirect effect); ADE average direct effect; TE total effect; Prop. Mediated proportion mediated. Please note that the mediator models for PA and NA are identical, but are displayed twice above each outcome model for reasons of overview. The estimates for the covariates are not displayed.
